# Effects of T2 Heat Treatment on Microstructure and Properties of the Selective Laser Melted Aluminum Alloy Samples

**DOI:** 10.3390/ma11010066

**Published:** 2018-01-03

**Authors:** Lianfeng Wang, Jing Sun, Xiaogang Zhu, Lingyu Cheng, Yun Shi, Lijie Guo, Biao Yan

**Affiliations:** 1 School of Materials Science and Engineering, Tongji University, Shanghai 201804, China; 2 Shanghai Aerospace Equipments Manufacturer Co., Ltd., Shanghai 200245, China; 15216615475@163.com (J.S.); xiaogangzhu1986@163.com (X.Z.); cly0129@126.com (L.C.); shiyun2009211@163.com (Y.S.); guolijie149@163.com (L.G.); 3 Shanghai Research Center of Complex Metal Parts by Additive Manufacturing, Shanghai 200245, China

**Keywords:** selective laser melting, aluminum alloy, T2 heat treatment

## Abstract

In this paper, aluminum alloy samples were fabricated by selective laser melting (SLM) and subsequently T2 heat treatment was undertaken. In order to obtain comprehensive results, various experiments on densification, hardness, tensile strength, bending strength and microstructure characterization were carried out. The results show that densification of samples after T2 heat treatment does not vary very much from the SLMed ones, while the Brinell hardness and strength decreases to about 50%. Moreover, the plasticity and fracture deflection increases about 3 fold. The effects on the microstructure and the mechanical properties of the SLMed aluminum alloy samples and subsequent T2 heat treatment were studied.

## 1. Introduction

Selective laser melting (SLM) is a new type of rapid prototyping technology that appeared in the 1990s. In the SLM process, a high-energy laser beam selectively melts metal powder completely and then the melted area metal rapidly cools and solidifies to form metal parts with high densification and precision. There are quite a few advantages of the SLM process, such as design freedom, time reduction, cost saving and structure strength etc.

In recent years, many experiments have been conducted on the preparation of titanium alloy, cobalt chromium alloy and iron-based alloy by SLM. Chlebus et al. studied the fabrication of Ti-Al-Nb alloy by SLM and suggested that by improving the process parameters, the titanium alloy parts exhibit better performance than traditionally processed components [1]. Kempen et al. researched the morphology, mechanical properties and the influence of heat treatment on the mechanical properties of SLM Ni-Fe alloys, demonstrating that the mechanical properties of SLM Ni-Fe alloy are obviously superior to traditionally wrought ones [2]. Zhai successfully fabricated Al_2_O_3_-TiN composites via a novel ball milling + addictive sintering process with the electrical percolation threshold value decreasing from 29% to 15% [3]. Ye prepared the mesoporous bioactive glass functionalized Ti-6Al-4V scaffolds by means of selective laser melting, attaining the controllable geometric features and preferable mechanical properties [4].

Compared with titanium alloy, cobalt chromium alloy and iron-based alloy, studies on SLM aluminum alloy are relatively late. Prior to 2011, there were very limited reports regarding SLM aluminum alloy. With the continuous development of SLM equipment, some research institutes have gradually carried out studies on SLM alloy [5,6,7,8,9,10]. Cai et al. described the application of the non-destructive X-ray-computed tomography (XCT) method to characterize the internal structure and enhance the understanding of the process parameters on material porosity and thus provided quality control of the SLM AlSi10Mg parts [11]. Salmi et al. used the semi-destructive hole-drilling method to measure the residual stresses on AlSi10Mg parts fabricated by an SLM process [12]. Lam et al. characterized microstructural features of AlSi10Mg samples produced by SLM [13]. Wang et al. and Trevisen et al. studied selective laser melted AlSi10Mg, including the process, the microstructure and the tensile strength [14,15]. The research of Trevisen et al. shows a freedom fabrication coupled with high mechanical properties related to a very fine microstructure [15]. Studies on SLM aluminum alloy provide the necessary experimental and theoretical basis for the large-scale application of the SLM technology in the manufacturing of aluminum alloy parts.

Based on the process characteristic of rapid heating and cooling, there is large inner stress in the SLM samples. As the inner stress increases, the samples will deform and even crack, resulting in the dramatic degeneration of the material performance which conforms to research by Hitzler [16]. Hitzler studied the anisotropy of the mechanical properties of stainless steel prepared by selective laser melting. Furthermore, the SLM samples exhibit severe anisotropy and poor plasticity as the grains grow under unidirectional heat transfer. To eliminate these effects, SLM samples require heat treatment. However, there is no standard heat treatment process for the samples. In this paper, the SLM aluminum samples were processed by T2 heat treatment. The densifications, hardness, tensile strength, bending strength and microstructure characterization were tested.

## 2. Experimental

### 2.1. Materials and SLM Process

**Figure 1:**
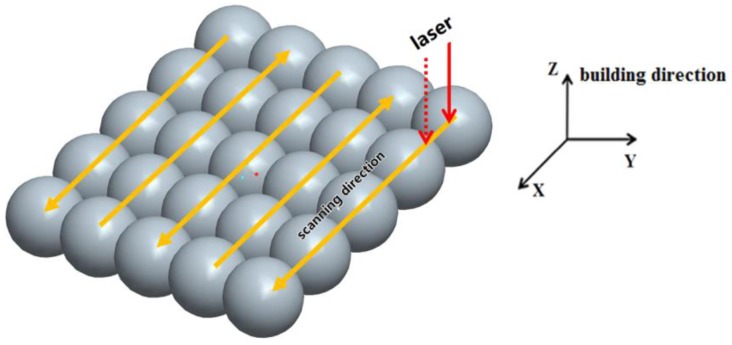
Schematic diagram of SLM processing, where the *X*- and *Y*-axis orientated parallel and normal to the building direction, *Z*-axis, of the SLM.

Gas-atomized AlSi10Mg powder with the particle distribution range of 20~60 μm was used as the raw material for the SLM process. All the samples were produced on the Renishaw (Wotton-under-Edge, UK) AM250 machine, equipped with a 400 W fiber laser beam. Samples were fabricated using laser power of 400 W, scanning speed of 1000 mm/s, hatching space of 175 μm and a layer thickness of 25μm. Layers were scanned by chessboard strategy with the 5 × 5 mm^2^ cubes and the rotation of 67° between the adjacent layers. The directly formed samples without any heat treatment were marked as SLMed samples as built. Figure 1 shows that the samples have their *X*- and *Y*-axis orientated parallel and normal to the building direction of the SLM. In the cross-section of the samples, the powder was set as the X-Y plane exposure to the laser source.

### 2.2. T2 Heat Treatment Process

The main purpose of T2 treatments to eliminate internal stress, stabilize dimension size and improve the plasticity of the samples. The specific parameters of T2 heat treatment for the SLMed AlSi10Mg samples are: heating up to 380 °C, holding for 45 min and then air cooling.

### 2.3. Mechanical Tests

After the T2 heat treatment, mechanical tests of the samples parallel and normal to the building direction of the SLM were done. For a convenient comparison, the testing conditions of T2 heat-treated samples are the same as those made by SLM. The density was tested according to Archimedes principle of drain age. The hardness test was performed according to the standard EN IOS 6506-1. The tensile test was examined based on the national standard GB/T 228-2002. The bending test was evaluated according to national standard YB/T 5349-2014.

### 2.4. Microstructure Characterization

To study the microstructure, the samples were sectioned, ground and polished using the standard metallographic procedures. Afterwards, the samples were etched using a solution containing 10% HF, 5% HNO_3_ and 85% distilled water (volume fraction) and investigated using a Zeiss Axiovert 200 optical microscope (Zeiss, Oberkochen, Germany) a Nova NanoSEM 450 scanning microscope(FEI, Brno, Czech Republic).

## 3. Results and Discussion

### 3.1. Effects of T2 Heat Treatment on the Densifications of the SLMed Samples

**Figure 2:**
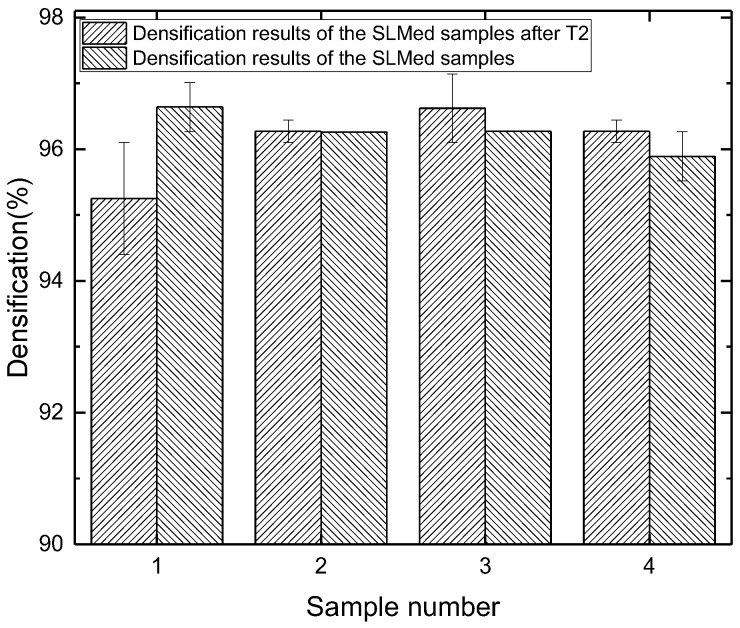
Densification results of the SLM samples.

The densifications of the SLMed samples and T2 heat-treated samples are shown in Figure 2. The densifications of all the T2 heat-treated samples are above 95% and the average densification is 96.1%. There is no difference of densification before and after the T2 heat treatment. The result indicates the T2 heat treatment has almost no influence on the densification of the SLMed aluminum alloy samples.

### 3.2. Effects of T2 Heat Treatment on the Hardness of the SLMed Samples

**Figure 3:**
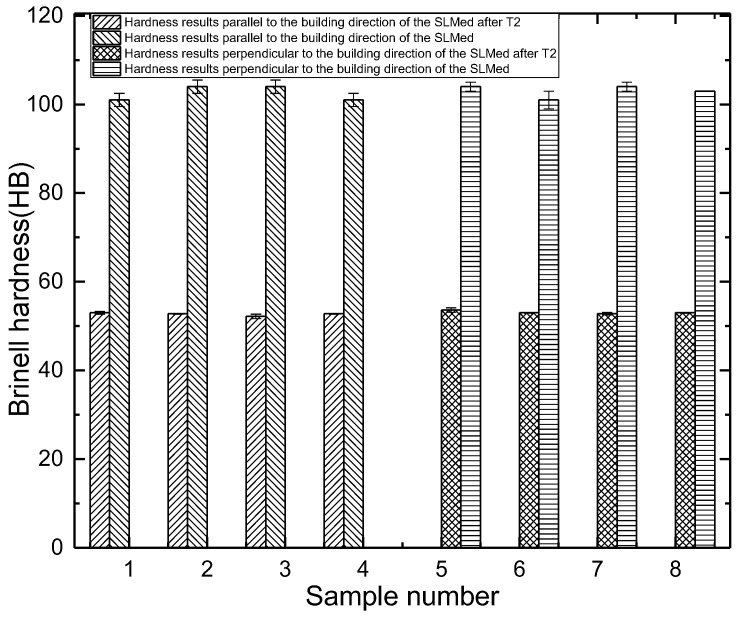
Hardness results of the SLMed samples after T2 heat treatment.

The Brinell hardness results of the samples treated by T2 heat treatment are shown in Figure 3. The average Brinell hardness of the T2 heat-treated samples parallel to the building direction of the SLM is 52.7 HB, and perpendicular to the building direction of the SLM is 53.1 HB. After T2 heat treatment, the Brinell hardness decreases to about 50% of the SLMed samples. In the SLM process, the extremely high cooling rate and temperature gradient leads to great thermal stress in the samples. Correspondingly, residual internal stress is formed in the samples. The existence of residual internal stress greatly improves the ability of samples to resist external load. Therefore, the hardness of the samples decreases significantly after annealing.

### 3.3. Effects of T2 Heat Treatment on the Tensile of the SLMed Samples

**Figure 4:**
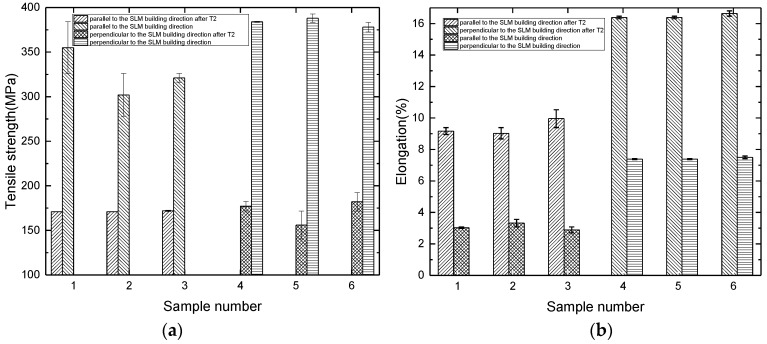
Tensile results of the SLMed samples after T2 heat treatment. (**a**) Tensile strength; (**b**) Elongation.

The tensile results of samples treated by T2 heat treatment are shown in Figure 4. Parallel to the building direction of the SLM, tensile strength is 171.3 MPa, yield strength is 105.7 MPa, and elongation is 9.4%. Compared with the SLMed samples, the strength decreases about 50%, and the plasticity increases about 3 times.

Perpendicular to the building direction of the SLM, tensile strength is 171.7 MPa, which is similar to that of the samples parallel to the building direction of the SLM. The elongation is 16.5%, which is nearly 2 times of that of the samples parallel to the building direction of the SLM. Compared with the SLMed samples, the strength decreases about 50%, and the plasticity increases about 2.5 times.

After T2 heat treatment, the strength of the samples clearly decreased, and the plasticity is clearly improved. During the SLM process, the melt rapidly forms and solidifies, and large internal stress accumulates in the samples. T2 heat treatment largely eliminates the residual internal stress, resulting in a decrease in the strength and increase in the plasticity of the samples. After T2 heat treatment, the difference of the strength and plasticity of the samples parallel and perpendicular to the building direction of the SLM decreases, and the degree of anisotropy becomes smaller.

**Figure 5:**
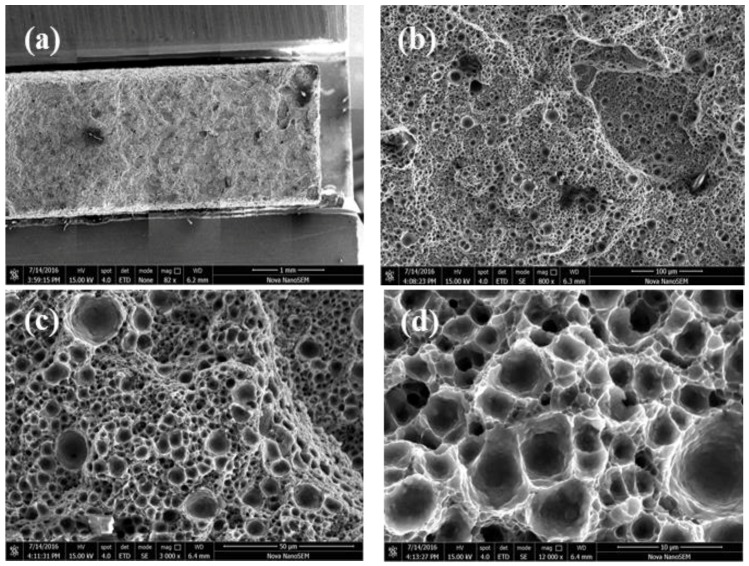
Tensile fracture surfaces of samples after T2 heat treatment: (**a**) the overall morphology of the tensile fracture; (**b**) the dimples are uniformly distributed on the fracture; (**c**) illustrated that the slope area in the fracture surface also be strews dimples indicating the good plasticity; (**d**) the dimple is polygonal and deep.

After T2 heat treatment, the tensile fracture surface of the sample shown in Figure 5a is the overall morphology. The overall fracture is flat, and there is a low fracture step. In Figure 5b, the dimples are uniformly distributed on the fracture, and the size of dimples is uniform. In Figure 5c, it is illustrated that the slope area in the fracture surface also be strews dimples indicating good plasticity. Furthermore, at high magnification (Figure 5d), the dimple is polygonal and deep, and the wall is thin and rough with a lamellar morphology.

**Figure 6:**
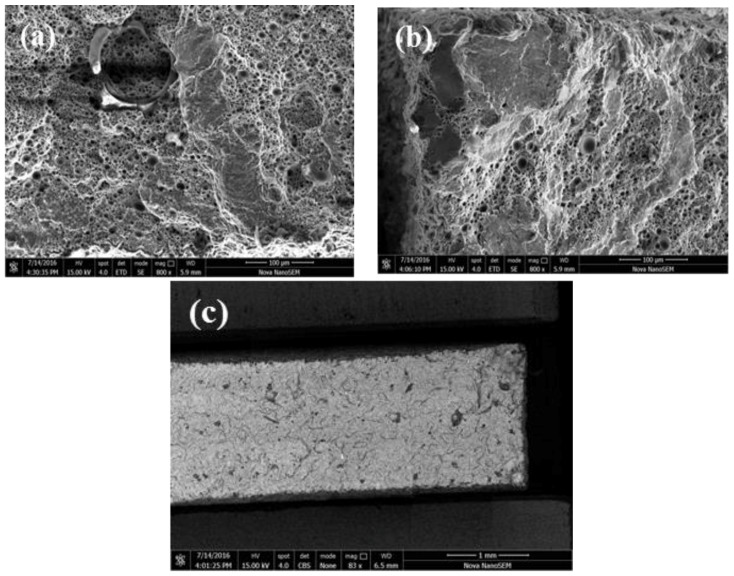
The quasi cleavage region on the tensile fracture surface of samples after T2 heat treatment: (**a**) an oxide layer around the hole on the fracture surface; (**b**) the quasi cleavage plane is flat; (**c**) the backscattering image of the fracture.

As shown in Figure 6, the island quasi cleavage fracture zone is distributed on the fracture surface of samples after T2 heat treatment, and the fracture morphology shows that the samples are ductile fractured. As shown in Figure 6a, there is an oxide layer around the hole on the fracture surface of samples. As shown in Figure 6b, the quasi cleavage plane is flat because the sample splits along the specific crystal surface. Meanwhile it is also found that non-melted particles are on the fracture, distributed around the cracks. Figure 6c is the backscattering image of the fracture. Compared with the SLMed samples, the dark oxides have a tendency of aggregation distribution, some are spherical distribution, and some are strip distribution. The oxides are banded in the fracture surface of the SLMed samples.

### 3.4. Effects of T2 Heat Treatment on the Bending of the SLMed Samples

The bending results of the SLMed samples parallel to the building direction of the SLM indicates that the average bending strength is 600 MPa, the prescribed non-proportional bending strength is 345 MPa, and the fracture deflection is 6.5 mm. Perpendicular to the building direction of the SLM, the average bending strength is about 660 MPa, the prescribed non-proportional bending strength is about 365 MPa, and the fracture deflection is 10 mm.

**Figure 7:**
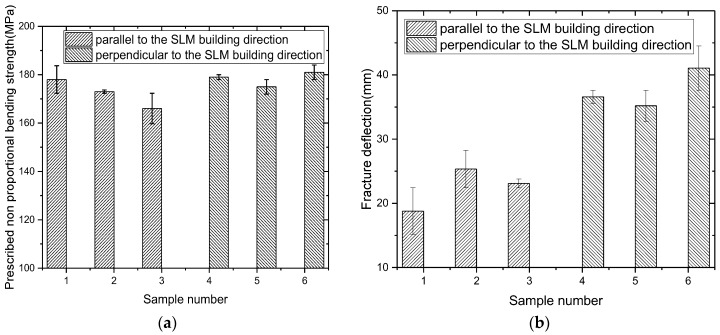
Bending results after T2 heat treatment. (**a**) Prescribed non proportional bending strength; (**b**) Fracture deflection.

The bending results of T2 heat-treated samples after T2 heat treatment are shown in Figure 7. Parallel to the building direction of the SLM, the average bending strength is 363.3 MPa, the prescribed non-proportional bending strength is 172.3 MPa, and the fracture deflection is 22.4 mm. Compared with the SLMed samples, the strength decreases about 39%, and the fracture deflection increases about 2.4 fold.

Perpendicular to the building direction of the SLM, the average bending strength is 366.7 MPa, the prescribed non-proportional bending strength is 178 MPa, and the fracture deflection is 37.6 mm. Compared with the SLMed samples, the strength decreases about 44%, and the fracture deflection increases about 2.7 times.

The results show that the texture of the samples after T2 heat treatment is obviously weakened. The samples restore and recrystallize, which leads to the decrease in strength and increase in plasticity. The difference of strength and plasticity between the different directions decreases. The above results are consistent with the tensile results.

**Figure 8:**
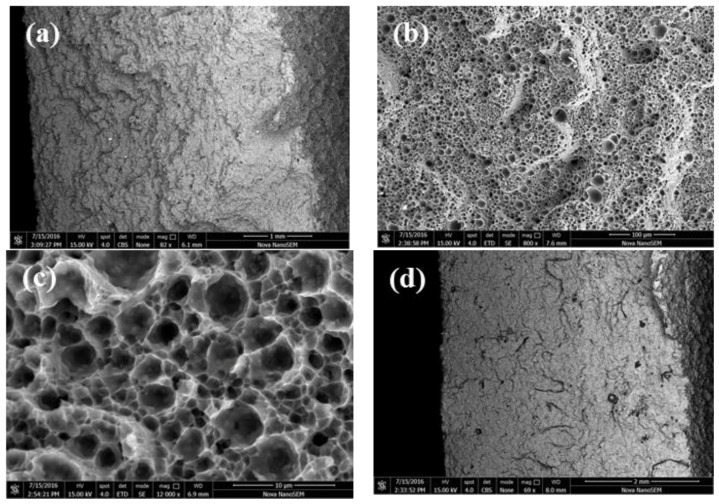
The SEM images of bending fracture of samples after T2 heat treatment: (**a**) the whole fracture morphology, and the fracture is flat with layered steps; (**b**) the dimples with uniform distribution on the fracture surface are observed at larger multiples; (**c**) the dimples are polygonal structures; (**d**) the backscattering image of the fracture.

The bending fracture of the samples after T2 heat treatment is shown in Figure 8. Figure 8a is the whole fracture morphology, and the fracture is flat with layered steps. As shown in Figure 8b, the dimples with uniform distribution on the fracture surface are observed at larger multiples, and the cleavage fracture is relatively smooth at the fracture step. As shown in Figure 8c, the dimples are polygonal structures. Compared with the SLMed samples, the uniformity of the dimple size is improved obviously. Figure 8d is the backscattering image of the fracture. The dark oxides are banded and tends to distribute at the fracture step.

### 3.5. Effects of T2 Heat Treatment on the Microstructure of the SLMed Samples

**Figure 9:**
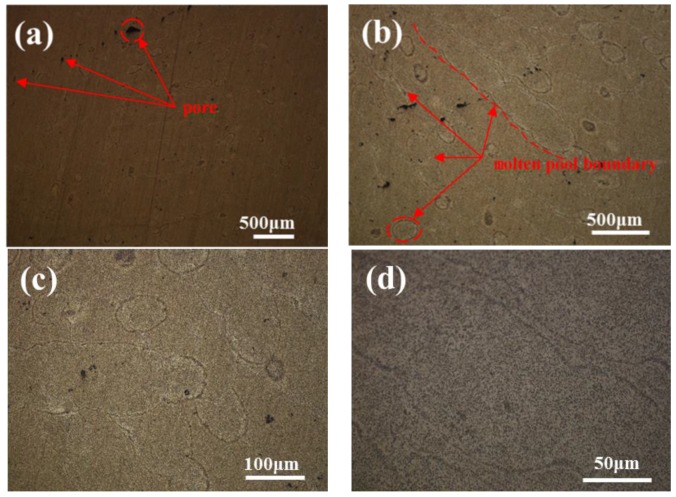
The optical microstructure of the samples perpendicular to the building direction of the SLM after T2 heat treatment: (**a**) the overall morphology; (**b**,**c**) found that the elliptical area of the precipitate accumulation in the overlapping area of the molten pool is more obvious: (**d**) the overlapping area of the molten pool is obviously depressed after corrosion.

The optical microstructure of the samples perpendicular to the building direction of the SLM after T2 heat treatment is shown in Figure 9. Figure 9a is the overall morphology. The boundary of the molten pool is fuzzy. After corrosion, in Figure 9b,c, it is found that the elliptical area of the precipitate accumulation in the overlapping area of the molten pool is more obvious. After the enlargement, as shown in Figure 9d, the overlapping area of the molten pool is obviously depressed after corrosion. In the overlapping area of the molten pool, a wider precipitate accumulation zone with a width of about 10 μm is found.

**Figure 10:**
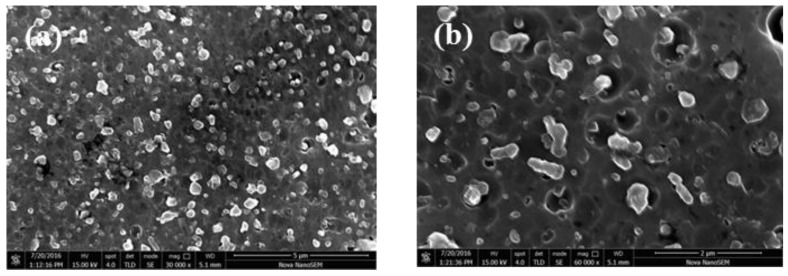
SEM images of the samples perpendicular to the building direction of the SLM after T2 heat treatment: (**a**) after T2 heat treatment, Si particles are fine and granular, and no large flake Si exists; (**b**) after spheroid, the size of Si particles decreases to nanometer scale, about 500 nm, and uniformly distributes in Al matrix.

The SEM images of the samples perpendicular to the building direction of the SLM after T2 heat treatment are shown in Figure 10. After T2 heat treatment, Si particles are fine and granular, and no large flake Si exists. We observed carefully and found that Si is basically completely spherical and edges and corners disappear, which is very important for improving the plasticity of the materials. After becoming spheroid, the size of the Si particles decreases to nanometer scale, about 500 nm, and uniformly distributes in the Al matrix. The number of Si increases, and the volume is significantly small.

**Figure 11:**
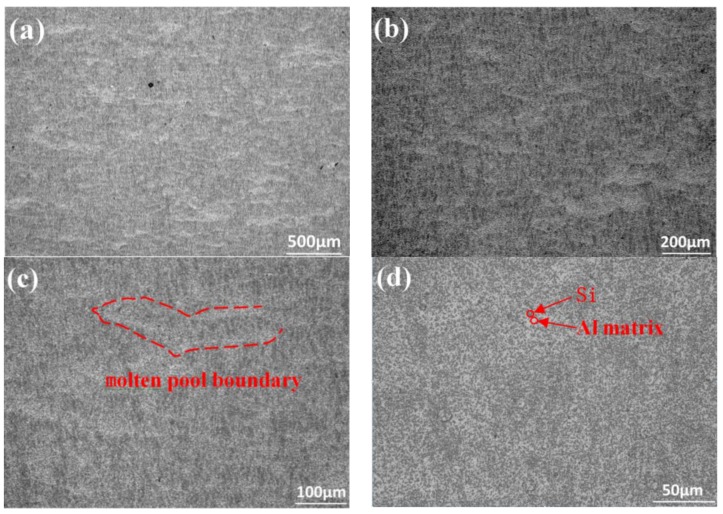
The optical microstructure of the samples parallel to the building direction of the SLM after T2 heat treatment: (**a**) the overall morphology; (**b**,**c**) the crescent shaped molten pool morphology is observed; (**d**) the columnar crystal growing through the molten pool.

The optical microstructure of the samples parallel to the building direction of the SLM after T2 heat treatment is shown in Figure 11. Figure 11a is the overall morphology. There are small holes distributed, but no obvious defect is found. After the enlargement, as shown in Figure 11b,c, the crescent shaped molten pool morphology is observed. The molten pool boundary is blurred, and the overlap area between the molten pools is clearly narrowed. After corrosion, it is found that the overlapping area of the molten pool is easily corroded, and slightly concave compared with the center of the molten pool. The point precipitation phase distributes uniformly in the matrix. The columnar crystal which passes through the molten pool boundary disappears as illustrated in Figure 11d. The columnar crystal growing through the molten pool is the reason why the strength of samples parallel to the building direction of the SLM is higher than that of samples perpendicular to the building direction of the SLM. The growth of columnar crystals requires directional emission of heat, and the cooling rate needs to be large. This growth mode leads to larger internal stress in columnar crystals, which shows an unstable state. T2 heat treatment leads to the transformation of columnar crystals into equiaxed grains. Therefore, after T2 heat treatment, the difference of properties of the samples decreases in the directions parallel and perpendicular to the building direction of the SLM.

**Figure 12:**
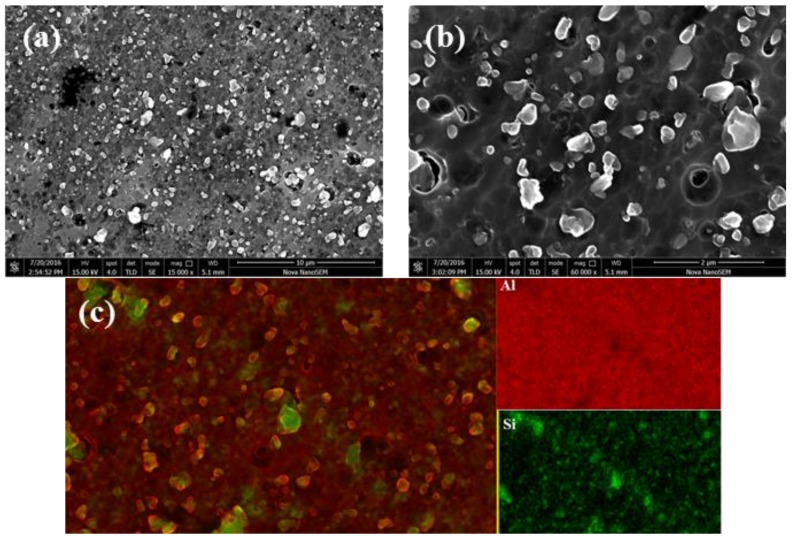
SEM images and elemental analysis of the samples parallel to the building direction of the SLM after T2 heat treatment. (**a**,**b**) the spheroidization of Si is uniformly distributed in the Al matrix, and the Si size reaches the nanometer scale; (**c**) the surface scanning results indicate that the white particles are high purity Si particles and are embedded in the Al matrix.

The SEM images and elemental analysis of the samples parallel to the building direction of the SLM after T2 heat treatment are shown in Figure 12. Similar to the results of the samples perpendicular to the building direction of the SLM after T2 heat treatment, the spheroidization of Si is uniformly distributed in the Al matrix, and the Si size reaches the nanometer scale. The surface scanning results shown in Figure 12c indicate that the white particles are high purity Si particles and are embedded in the Al matrix. In the SLMed samples, the surface scanning cannot clearly separate the Si from the matrix due to the small difference in the concentration of Si in the matrix between the particles.

The results show that during the T2 heat treatment, the solid solution of Si in the matrix has enough time to diffuse, precipitate, absorb on the original Si crystal nucleus building a new crystal nucleus and grow into a new particle. The amount of Si particles increases and the volume decreases in the samples after T2 heat treatment. 

T2 heat treatment is a stress relief annealing, which promotes the homogenization and eliminates the internal stress of the samples. After T2 heat treatment, the boundary of molten pool disappears and becomes vague. The precipitates at the molten pool boundary dissolves in the matrix, and the microstructure of the samples is homogenized. In the process of the SLM, supersaturated solid solution is formed due to non-equilibrium solidification, and the solubility of Si in Al matrix can reach as high as 10%. This results in a large amount of supersaturated Si elements precipitates and forms a large amount of dispersed Si phase during the T2 heat treatment. Si is uniformly distributed in the matrix, and there is no obvious trend of distribution along the grain boundary, which is beneficial to the plasticity rise of the samples. However, the yield strength and tensile strength of the samples are greatly reduced when the grain boundary pinning effect is lost.

## 4. Conclusions

The effects of T2 heat treatment on the densification, hardness, tensile properties, bending properties, and microstructure of SLMed aluminum alloy samples have been studied. The main conclusions are as follows:

(1) Compared with the SLMed samples, the densification is not very different. T2 heat treatment has no obvious influence on the densification of the SLMed samples.
(2) After T2 heat treatment, the Brinell hardness, tensile strength, and bending strength of the SLMed samples decrease about 50%. The plasticity and fracture deflection clearly increases, and the anisotropy decreases.
(3) The T2 heat treatment promotes the homogenization of elements, and the precipitates in the molten pool boundary dissolves in the matrix. The samples undergo recrystallization, Si particle size grows and tends to form spherical. The internal stress of the samples is effectively released.
